# P-762. Prevalence and/or Recurrence of Non-Tubercular Mycobacterial Pulmonary Disease in Structural and Airway Lung Disease

**DOI:** 10.1093/ofid/ofae631.957

**Published:** 2025-01-29

**Authors:** Mohit Chowdhury, Urvashi B Singh, Animesh Ray, Sanjeev Sinha

**Affiliations:** All India Institute of Medical Sciences, NEW DELHI, Delhi, India; All India Institute of Medical Sciences ,New Delhi, New Delhi, Delhi, India; All India Institute of Medical Sciences, New Delhi, India, New Delhi, Delhi, India; All India Institute of Medical Sciences, New Delhi, New Delhi, Delhi, India

## Abstract

**Background:**

Nontuberculous mycobacterial pulmonary disease (NTM-PD) frequently remains undetected, especially among patients with preexisting respiratory conditions who are prone to develop the disease. This study aims to explore the prevalence, species diversity, and outcomes of NTM-PD in India, an uncharted topic.

**Figure:**
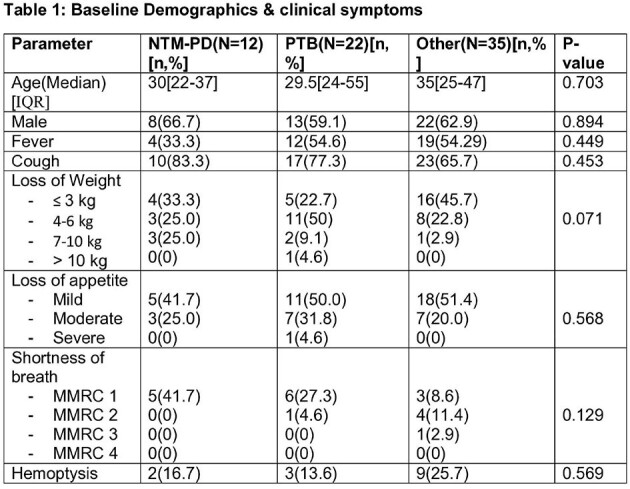

**Methods:**

We conducted a prospective observational study, enrolling 69 patients with symptomatic chronic respiratory diseases or a history of Tuberculosis (TB). These patients underwent thorough clinical assessment, chest computed tomography scans, and either a single bronchoscopic sample or three sputum samples for microbiological analysis, along with relevant biochemical investigations. Diagnosis of NTM-PD followed the ATS 2020 diagnostic criteria. The growth of the organism was compared using patented indigenous media with or without p-nitrobenzoic acid.

**Figure:**
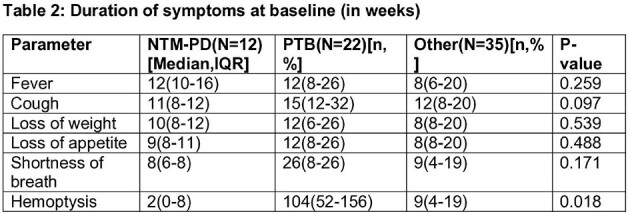

**Results:**

The prevalence of NTM-PD was 17.4%, with TB affecting 31.9% of cases. Baseline clinical symptoms and their duration showed no statistically significant differences, except NTM-PD patients with hemoptysis had significantly shorter symptom durations than other groups. Isolation rates were notably higher with the patented indigenous media compared to the mycobacterial growth indicator tube for both NTM (100% vs. 33.3%, p < 0.001) and *Mycobacterium tuberculosis* (M.Tb) (57.1% vs. 47.6%, p < 0.001). NTM-PD patients exhibited a significantly higher proportion of fibro-cavitary disease compared to other groups when comparing radiological findings. After monitoring NTM-PD patients following treatment, one patient (20%) experienced a recurrence of symptoms within six months, rest continue to be under follow-up.

**Figure:**
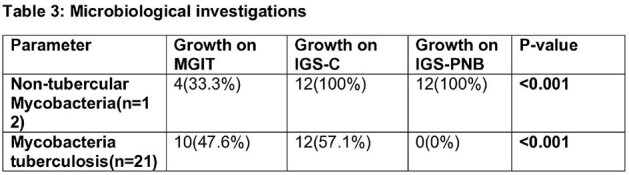

**Conclusion:**

In a high-burden TB setting, clinical suspicion and effort to look for NTM-PD is often low. Our study highlights the high prevalence of NTM-PD in individuals with chronic lung conditions/past history of TB. Patients with NTM-PD show higher rates of fibro-cavitary disease, possibly allowing the NTM to form biofilms and hence lead to relapses. Diagnosis of NTM-PD is thus important, to ensure desired treatment for the recommended duration and a thorough and prolonged post-treatment monitoring due to higher relapse risk.

**Figure:**
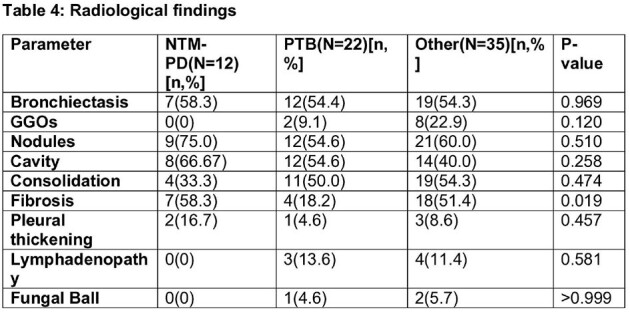

**Disclosures:**

**All Authors**: No reported disclosures

